# Urological applications of single-site laparoscopic surgery

**DOI:** 10.4103/0972-9941.72394

**Published:** 2011

**Authors:** Andrew Symes, Abhay Rane

**Affiliations:** Department of Urology, East Surrey Hospital, Redhill, U.K

**Keywords:** Laparoscopy, laparoendoscopic single-site surgery, robotic, single-port, single-site, urology

## Abstract

Single-port, single-incision laparoscopy is part of the natural development of minimally invasive surgery. Refinement and modification of laparoscopic instrumentation has resulted in a substantial increase in the use of laparoendoscopic single-site surgery (LESS) in urology over the past 2 years. Since the initial report of single-port nephrectomy in 2007, the majority of laparoscopic procedures in urology have been described with a single-site approach. This includes surgery on the adrenal, ureter, bladder, prostate, and testis, for both benign and malignant conditions. In this review, we describe the current clinical applications and results of LESS in Urological Surgery. To date this evidence comes from small case series in centres of excellence, with good results. Further well-designed prospective trials are awaited to validate these findings.

## INTRODUCTION

Natural orifice translumenal endoscopic surgery (NOTES) and laparoendoscopic single-site surgery (LESS) represent the latest evolution of minimally invasive surgery. They have been developed with the aim of preventing port-site complications, decreasing discomfort, and improving cosmetic outcomes associated with standard laparoscopic/robotic surgery.[1]

NOTES involves diagnostic or therapeutic interventions performed via existing orifices of the human body (mouth, anus, urethra, and vagina).[2] In 2002, Gettman *et al*.[3] reported the first successful experimental application of NOTES in urological surgery when they performed six laparoscopic transvaginal nephrectomies in a porcine model. Limitations in instrumentation, such as awkward shape and lack of mobility, and a less familiar working angle and operative approach, led to a steep learning curve with resultant prolonged operative times (210–360 min). Additionally, five of the six pigs required a separate transabdominal port for completion. Although NOTES might be a promising surgical approach, the current limitations have slowed its application in humans. The surgical difficulties encountered with NOTES, and the continued need for a transabdominal port, have led to an increasing interest in single-incision laparoscopy, where several instruments are inserted through a single abdominal or retroperitoneal incision. Several terms and acronyms have been used to refer to this technique until a consensus statement agreed to use the term laparoendoscopic single-site surgery or “LESS”.[4]

The first urological use of LESS was reported in 2007[5] with the completion of the first single-port nephrectomy for a small nonfunctioning kidney, as well as a transperitoneal ureterolithotomy, with a mean operative time of 90 min and no intraoperative complications. Since then, clinical success has been reported for a range of LESS surgical procedures in urology, and the concept of LESS has gained increasing popularity among urologists. In this review, we describe the current clinical applications and results of LESS in Urological Surgery.

## ADRENAL

Attempts to perform urological surgery through a single-incision began with a report by Hirano *et al* in 2005.[6] The investigators used a specialized resectoscope tube with a 4 cm diameter and standard laparoscopic instruments to perform a retroperitoneoscopic adrenalectomy. The average operating time was 203 min, the mean blood loss was 252 mL, and four patients received blood transfusions. Conversion was necessary in one case. Single-port laparoscopic transabdominal adrenalectomy has been performed successfully since 2008.[7,8] Desai *et al* reported mean operative times of 2 h, with estimated blood loss of 10–150 mL, and mean length of hospital stay 2.2 days.[8] A recent case–control study compared 50 single-site retroperitoneoscopic adrenalectomies (SARA) with 47 conventional retroperitoneoscopic adrenalectomies (CORA).[9] A single 2 cm skin incision was used below the tip of the 12th rib and a 10 mm cutting port (Visiport^®^, Covidien) used to enter the retroperitoneum. A 30° endoscope was subsequently used to bluntly develop the space with high CO_2_ pressures (20–30 mmHg). The 10 mm port was then replaced with two 5-mm ports through which a 5-mm endoscope and a 5-mm bipolar scissor were placed to complete the surgery. SARA was possible in 41 cases with similar complication rates between the two groups. While the mean operative times were significantly higher for SARA (56 vs 40 min), postoperative analgesic requirements and hospital length of stay were significantly less.

## KIDNEY

Since the first laparoscopic nephrectomy was performed in 1991,[10] laparoscopic renal surgery has evolved rapidly with improving techniques and technology, including robotics. Complex reconstructive, extirpative, and ablative operations are now possible, with the clinical benefits of reduced postoperative pain and hospital stay, with improved cosmesis and convalescence. Mirroring this single site renal surgery is also now possible, encompassing many of the techniques used in conventional open and laparoscopic surgery.

The first single-port, nontransumbilical, simple nephrectomy was presented at the World Congress of Endourology in 2007.[5] An R-port was situated in a flank incision for retroperitoneoscopy. The procedure was performed successfully and without complication. The first multitrocar single-incision transumbilical nephrectomy was performed successfully by Raman *et al*.[11] Three human nephrectomies were performed with a mean operative time of 133 min. Two were for benign indications and the third for a 4.5 cm clear cell renal cell carcinoma. Rather than a single port, three conventional trocars were placed through a single umbilical incision, and an additional port was required only for a right nephrectomy to provide liver retraction. The first single-port transumbilical nephrectomy followed shortly using the R-port and specialized curved laparoscopic graspers.[8] A simple nephrectomy was performed in over 3 h without the need for any other incisions.

LESS radical nephrectomy has been described in several studies. Ponsky *et al*[12] described the first LESS radical nephrectomy performed with three standard ports placed through a Gelport^®^. This was located in a 7 cm paramedian incision and operating time was 96 min. There were no complications and the specimen was retrieved intact.

Stolzenburg *et al*[13] have published their experience with LESS radical nephrectomy. The Triport^®^ device was used through a single transumbilical incision. All nephrectomies were performed successfully without the need for conversion to conventional laparoscopic surgery, and without intra- or postoperative complications. Their median operative times were 141 min, and mean blood loss was 103 mL. They subsequently published their technique in detail, documenting that while this technique is indeed possible, it is challenging requiring high levels of laparoscopic skill.[14]

Nephron sparing surgery (NSS) has been shown consistently to have similar oncological outcomes for small renal tumours (<7 cm) as radical nephrectomy.[15,16] NSS may result in better renal functional outcomes, translating into improved overall long-term survival.[17] The American Urological Association advocate open partial nephrectomy as the standard of care for the treatment of small renal masses, but they also report that patients with small peripheral lesions meeting the criteria for open partial nephrectomy should be considered for laparoscopic partial nephrectomy. Laparoscopic partial nephrectomy is challenging, and with single-site surgery it is even more so. Only small series exist and thus it is impossible to extrapolate overall outcomes. Aron *et al*[18] published on four LESS partial nephrectomies using the Triport^®^. An additional 5-mm grasper was used in one case and the mean tumour size was 3 cm. Operating times were long (median 270 min), and average warm ischemia time was 20 min (11–29 min). All the margins were negative and there were no intraoperative complications. One patient had postoperative bleeding and pulmonary embolism. Kaouk *et al*[19] recently published on seven LESS partial nephrectomies, two of which utilized robotic assistance. A single multichannel port, or Triport^®^ provided intraabdominal access. One patient required conversion to conventional laparoscopy due to intraoperative bleeding. There was one positive margin but no postoperative complications.

The two largest urological LESS series to date reported on 200 procedures.[20,21] Included in them are a large number of extirpative renal procedures. Desai *et al*[20] reported on 23 simple (n = 14), radical (3) and partial nephrectomies (6). The mean operative times were 145, 208, and 271 min and the estimated blood loss was 109, 200, and 475 mL, respectively. There was one conversion to conventional laparoscopy in the partial nephrectomy group, all of which required an additional port. One of these patients required embolization for postoperative haemorrhage, and there were no positive margins. White *et al*[21] performed 28 simple (n = 7), radical (6) and partial nephrectomies 15). The mean operative times were 156, 206, 196 min and the estimated blood loss was 121, 146, and 422 mL, respectively. Four patients required transfusion post partial nephrectomy and there were two conversions in this group, along with one positive margin.

Cryoablation of renal masses offers an alternative to open or laparoscopic nephron sparing surgery. Standard laparoscopic technique utilizes three ports either trans- or retroperitoneally. Goel *et al*[22] successfully performed six single-port renal cryoablations using the Uni-X™ single port access device (Pnavel systems, NJ, USA). Two cases were performed transperitoneally through the umbilicus, with the rest performed retroperitoneally (placement below the 12th rib). The cryoprobe and ultrasound probe for monitoring the iceball were placed through the single port. For tumours greater than 2 cm in size, an additional percutaneously inserted cryoprobe was used. The mean operative time was 170 min and the length of hospital stay was 2.3 days, and there were no intraoperative complications.

Nine cases of LESS nephroureterectomy were included in the two large LESS series.[20,21] Cystoscopic resection or a laparoscopic stapling device was used to manage the distal ureter. Seo *et al*[23] used a homemade single-port device to successfully complete four LESS nephroureterectomies. Either an open resection of the bladder cuff through a separate open incision or a laparoscopic stapler was used to manage the distal ureter. Ponsky *et al*[24] performed a radical nephrectomy and nephroureterectomy successfully through a 7.5 cm pfannensteil incision using the Gelport^®^ device. This allowed open access to the distal ureter but bariatric instruments were required for length.

LESS transumbilical live donor nephrectomy was first reported by Gill *et al*,[25] using a transumbilical R-port^®^ for access. The procedure was successful in all four patients with a median operating time of 3.3 h and minimal blood loss. The median warm ischaemia times were only 6.2 min, and the length of the harvested renal artery, vein, and ureter was excellent. There were no complications and all the allografts functioned immediately on transplantation. A subsequent matched-pair analysis comparing LESS donor nephrectomy (LESS-DN) with standard laparoscopic donor nephrectomy (LLDN) showed equivalence in operating time, blood loss, and hospital stay.[26] The mean warm ischaemia time was longer in the LESS-DN group (3 vs 6.1 min: *P* < 0.0001), however, allograft function was comparable between groups. The patient convalescence after discharge (oral analgesia requirements, days off work, and days to 100% physical, recovery) was significantly faster in the LESS-DN group. The two largest series to report on urological LESS surgery include 17[20] and 19[21] LESS donor nephrectomies. The median warm ischaemia times were 5.9 and 5.29 min, respectively. There was one graft loss on postoperative day 1, secondary to intravascular clotting of uncertain aetiology.

LESS pyeloplasty for pelvi-ureteric junction obstruction has been reported on with seemingly good results. Desai *et al*[20] performed 17 pyeloplasties, two with robotic assistance. The mean operative time and blood loss were 236 min and 79 mL, respectively. There were no complications, but all cases required an additional 2-mm port to aid suturing. One case was converted to conventional laparoscopy. All patients were symptom-free post procedure and postoperative imaging showed unobstructed drainage in 15 of the 16 patients in whom data were available. The authors felt that pyeloplasty was ideally suited to LESS, given the good outcomes, the importance of cosmesis in this often young patient group, and the ability to deal with crossing vessels and concomitant stones at the time of surgery. White *et al*[21] performed eight pyeloplasties, one with the aid of the Da Vinci robotic platform. The mean operative time and blood loss were 233 min and 62.5 mL, respectively. Renographic follow-up was documented as within normal limits and there were no complications apart from a wound site hernia.

Other LESS renal procedures reported are renal cyst excision,[20] renal biopsy,[21] and renal cyst decortication.[20]

## URETER

Despite its high median stone-free rate, open ureterolithotomy is no longer recommended as a first-line treatment for ureteric stones, because of longer hospitalization and greater postoperative morbidity. Ureterolithotomy does remain a primary or salvage option in difficult situations with large, impacted or multiple ureteric stones. Laparoscopic ureterolithotomy is preferable because of its decreased invasiveness, and resultant reduced morbidity, and has been well described in this setting.[27] One case of LESS ureterolithotomy was successfully performed in the first series of LESS urological surgery.[5] Subsequently, Kim *et al*[28] performed seven single-site transumbilical laparoscopic ureterolithotomies using a homemade port. The mean stone diameter was 21.9 mm (range 16–27 mm), with mean operative time of 197.1 min. The average length of stay was 3 days, and there were no major complications documented. Six patients had their stones removed completely, with the seventh reported as having clinically insignificant fragments (<4 mm) that migrated to the kidney and were subsequently spontaneously passed. No strictures developed on intravenous urography follow-up.

Desai *et al*[20] performed two ureteroneocystostomies and three ileal interpositions for ureteric strictures using a laparoscopic single-site approach. All showed unobstructed drainage on follow-up. White *et al*[21] performed one ureteric reimplantation into the bladder successfully without complication.

Autorino *et al*[29] reported this year on the first case of retrocaval ureter managed by LESS surgery. A transumbilical approach was used with mobilization of the entire ureter. This was then transected and relocated lateral to the inferior vena cava. A direct spatulated re-anastomosis was performed over a ureteric stent. At 3 months, all the patients were symptom free and unobstructed on diuretic renography.

## BLADDER

Radical cystectomy with bilateral pelvic lymph node dissection is the standard of care for patients with muscle invasive urothelial carcinoma of the bladder. It is also an option for patients in whom conservative treatment has failed in high-risk nonmuscle invasive bladder cancer. There is, however, significant morbidity associated with it, and this has led to the development of laparoscopic and robotic approaches. Although long-term data are lacking, in the short term at least laparoscopic (including robotic-assisted) radical cystectomy appears to offer equivalent oncological control, with reduced blood loss and analgesic requirements as well quicker return to bowel function.[30] Kaouk *et al*[31] have recently published the first series assessing the feasibility and outcomes of laparoscopic radical cystectomy and pelvic node dissection through a single umbilical port. Three patients were successfully operated on, and underwent extracorporeal urinary diversion by way of extension of the umbilical port site. The mean operative time was 315 min (not including conduit formation) and there was minimal blood loss (average 217 mL). All margins and nodes were negative with the mean node yield 16. Average length of stay was 6 days and at 2 years all patients were disease free. The authors conclude that this technique is feasible but that randomized prospective studies are needed to establish its place.

## PROSTATE

Minimally invasive radical prostatectomy is fast becoming the standard of care for prostate cancer. Robotic-assisted laparoscopic prostatectomy using the Da Vinci surgical system (Intuitive Surgical, Sunnyvale, CA, USA) is widespread, and although randomized prospective studies do not exist, clinical outcomes seem to be excellent in high volume centres. LESS radical prostatectomy was first described in 2008 by Kaouk *et al*.[32] Four patients with clinical stage T1c prostate cancer, no history of pelvic surgery, and BMI < 35 kg/m^2^ were operated on through the umbilicus using a single three-channel port. The urethrovesical anastomosis was performed with interrupted sutures and extracorporeal knot tying. The mean operative time was 285 min and the mean blood loss 288 mL. The average length of stay was 2.5 days and there were no intraoperative complications. One patient, however, developed a recto-urethral fistula. Short-term oncological and functional results were equivalent to other laparoscopic series. The same series was extended to six patients subsequently, with three having positive margins but remaining biochemically free of disease.[21]

Kaouk *et al*. reported, in 2009, the first successful series of single-port robotic procedures in humans, including radical prostatectomy.[33] A robotic 12-mm scope and 5-mm grasper were introduced through a multichannel single port (R-Port), while an additional 5- or 8-mm robotic port was introduced through the same umbilical incision (2 cm) alongside the multichannel port to facilitate entry of robotic instruments.

The authors noted an improved facility for intracorporeal dissecting and suturing due to robotic instrument articulation and stability.

Together with their preliminary experience in a cadaver model, Barret *et al*. also reported their experience with a *hybrid* LESS robotic-assisted radical prostatectomy in a single patient.[34] They placed two 8-mm robotic ports and a 12-mm port for the robotic camera into a 4-cm umbilical incision. An additional 5-mm port was placed at the right lower abdomen. More recently, the same authors reported their initial case of a complete robotic LESS radical prostatectomy.[35] They utilized a single umbilical incision and placed a 12-mm port for the robotic scope, a 5-mm port for the assistant, and two 8-mm ports for the robotic arms arranged in a rhomboid fashion. No intraoperative complications occurred and the surgical margins were negative. Significant external robotic arm collisions were experienced as well as a reduced space for the assistant to work.

Desai *et al* published the initial series of single-port transvesical simple prostatectomy (STEP).[36] This series was extended in 2010 to include 34 patients with large volume BPH (>60 G).[37] Mean prostate volume was 102.5 mL. A single-port device (Triport^®^ or Quadport^®^) was inserted percutaneously into the bladder through a 2–3 cm incision in the suprapubic skin crease. The prostate adenoma was enucleated transvesically using standard laparoscopic instruments, and extracted in pieces through the port. Digital assistance was required in 55% of cases. One patient died in this series of uncontrolled haemorrhage, a Jehovah’s Witness who refused blood products. In addition, there was one bowel injury and two open conversions were necessary. Digital enucleation required extension of the incision by 1–2 cm. Average length of stay was 3 days, and at follow-up there was significant improvement of obstructive parameters with no patient catheter dependent or incontinent. One case in this series utilized the Da Vinci surgical platform. The authors commented that the Endowrist™ technology allowed better maneuverability in the transvesical environment. Specifically, the robotic grasper provided excellent retraction of the adenoma as it was progressively enucleated.

## OTHER LESS UROLOGICAL APPLICATIONS

Single-site laparoscopic surgery has been reported in small numbers for a variety of other urological conditions. Paediatric varicocoeles have been successfully operated open through a single transumbilical port.[38] A mesh sling has been successfully removed from the bladder via a transvesical approach.[20] Thirteen sacrocolpopexies were successfully performed without complication with a mean operative time of 182 min and an estimated blood loss of 46.9 mL.[21] One orchidopexy and one orchidectomy have been successfully performed through a single-incision without complication.[39]

## CONCLUSIONS

LESS in urology is in its relative infancy but to date has proven to be safe and feasible in the hands of experienced laparoscopic surgeons, using specially designed ports and instruments in selected patients. Further refinements in instrumentation and operative techniques will be required before this method of surgical access can be widely accepted. LESS in urology requires prospective randomized studies to define the benefits of this technique for patients, as well as to elucidate the cost-effectiveness of the approach.

The introduction of robotics into LESS may make these techniques finally realize their potential. Improvements in existing robotic platforms[40] may pave the way for their widespread use in LESS and possibly NOTES. In addition new robotic systems, including master–slave systems, flexible robots, in vivo miniature robots, or a combination therein might bring LESS/NOTES to its full potential in the future.[41,42]

## References

[1] Gettman MT, Box G, Averch T, Cadeddu JA, Cherullo E, Clayman RV (2008). Consensus statement on natural orifice transluminal endoscopic surgery and single-incision laparoscopic surgery: Heralding a new era in urology?. Eur Urol.

[2] Box G, Averch T, Cadeddu J, Cherullo E, Clayman R, Desai M (2008). Nomenclature of natural orifice translumenal endoscopic surgery (NOTES) and laparoendoscopic single-site surgery (LESS) procedures in urology. J Endourol.

[3] Gettman MT, Lotan Y, Napper CA, Cadeddu JA (2002). Transvaginal laparoscopic nephrectomy: Development and feasibility in the porcine model. Urology.

[4] Gill IS, Advincula AP, Aron M, Caddedu J, Canes D, Curcillo PG (2010). Consensus statement of the consortium for laparoendoscopic single-site surgery. Surg Endosc.

[5] Rane A, Rao P, Bonadio F, Rao P (2007). Single port laparoscopic nephrectomy using a novel laparoscopic port (R-port) and evolution of single laparoscopic port procedure (SLIPP). J Endourol.

[6] Hirano D, Minei S, Yamaguchi K, Yoshikawa T, Hachiya T, Yoshida T (2005). Retroperitoneoscopic adrenalectomy for adrenal tumors via a single large port. J Endourol.

[7] Castellucci SA, Curcillo PG, Ginsberg PC, Saba SC, Jaffe JS, Harmon JD (2008). Single port access adrenalectomy. J Endourol.

[8] Desai MM, Rao PP, Aron M, Pascal-Haber G, Desai MR, Mishra S (2008). Scarless single port transumbilical nephrectomy and pyeloplasty: First clinical report. BJU Int.

[9] Walz MK, Groeben H, Alesina PF (2010). Single-access retroperitoneoscopic adrenalectomy (SARA) versus conventional retroperitoneoscopic adrenalectomy (CORA): A case-control study. World J Surg.

[10] Clayman RV, Kavoussi LR, Soper NJ, Dierks SM, Meretyk S, Darcy MD (1991). Laparoscopic nephrectomy: Initial case report. J Urol.

[11] Raman JD, Bensalah K, Bagrodia A, Stern JM, Cadeddu JA (2007). Laboratory and clinical development of single keyhole umbilical nephrectomy. Urology.

[12] Ponsky LE, Cherullo EE, Sawyer M, Hartke D (2008). Single access site laparoscopic radical nephrectomy: Initial clinical experience. J Endourol.

[13] Stolzenburg JU, Hellawell G, Kallidonis P, Do M, Haefner T, Dietel A (2009). Laparoendoscopic single-site surgery: Early experience with tumor nephrectomy. J Endourol.

[14] Stolzenburg JU, Kallidonis P, Hellawell G, Do M, Haefner T, Dietel A (2009). Technique of laparoscopic-endoscopic single-site surgery radical nephrectomy. Eur Urol.

[15] Leibovich BC, Blute ML, Cheville JC, Lohse CM, Weaver AL, Zincke H (2004). Nephron sparing surgery for appropriately selected renal cell carcinoma between 4 and 7 cm results in outcome similar to radical nephrectomy. J Urol.

[16] Antonelli A, Cozzoli A, Nicolai M, Zani D, Zanotelli T, Perucchini L (2008). Nephron-sparing surgery versus radical nephrectomy in the treatment of intracapsular renal cell carcinoma up to 7cm. Eur Urol.

[17] Miller DC, Schonlau M, Litwin MS, Lai J, Saigal CS (2008). Renal and cardiovascular morbidity after partial or radical nephrectomy. Cancer.

[18] Aron M, Canes D, Desai MM, Haber GP, Kaouk JH, Gill IS (2009). Transumbilical single-port laparoscopic partial nephrectomy. BJU Int.

[19] Kaouk JH, Goel RK (2009). Single-port laparoscopic and robotic partial nephrectomy. Eur Urol.

[20] Desai MM, Berger AK, Brandina R, Aron M, Irwin BH, Canes D (2009). Laparoendoscopic single-site surgery: Initial hundred patients. Urology.

[21] White WM, Haber GP, Goel RK, Crouzet S, Stein RJ, Kaouk JH (2009). Single-port urological surgery: Single-center experience with the first 100 cases. Urology.

[22] Goel RK, Kaouk JH (2008). Single port access renal cryoablation (SPARC): A new approach. Eur Urol.

[23] Seo IY, Hong HM, Kang IS, Lee JW, Rim JS (2010). Early experience of laparoendoscopic single-site nephroureterectomy for upper urinary tract tumors. Korean J Urol.

[24] Ponsky LE, Steinway ML, Lengu IJ, Hartke DM, Vourganti S, Cherullo EE (2009). A Pfannenstiel single-site nephrectomy and nephroureterectomy: A practical application of laparoendoscopic single-site surgery. Urology.

[25] Gill IS, Canes D, Aron M, Haber GP, Goldfarb DA, Flechner S (2008). Single port transumbilical (E-NOTES) donor nephrectomy. J Urol.

[26] Canes D, Berger A, Aron M, Brandina R, Goldfarb DA, Shoskes D (2010). Laparo-endoscopic single site (LESS) versus standard laparoscopic left donor nephrectomy: Matched-pair comparison. Eur Urol.

[27] Goel A, Hemal AK (2001). Upper and mid-ureteric stones: A prospective unrandomized comparison of retroperitoneoscopic and open ureterolithotomy. BJU Int.

[28] Kim TH, Jeong BC, Seo SI, Jeon SS, Han DH (2010). Transumbilical laparoendoscopic single-site ureterolithotomy for large impacted ureteral stones: Initial experiences. Korean J Urol.

[29] Autorino R, Khanna R, White MA, Haber GP, Shah G, Kaouk JH (2010). Laparoendoscopic Single-site Repair of Retrocaval Ureter: First Case Report. Urology.

[30] Berger A, Aron M (2008). Laparoscopic radical cystectomy: Long-term outcomes. Curr Opin Urol.

[31] Kaouk JH, Goel RK, White MA, White WM, Autorino R, Haber GP (2010). Laparoendoscopic Single-site Radical Cystectomy and Pelvic Lymph Node Dissection: Initial Experience and 2-Year Follow-up. Urology.

[32] Kaouk JH, Goel RK, Haber GP, Crouzet S, Desai MM, Gill IS (2008). Single-port laparoscopic radical prostatectomy. Urology.

[33] Kaouk JH, Goel RK, Haber GP, Crouzet S, Stein RJ (2009). Robotic single-port transumbilical surgery in humans: Initial report. BJU Int.

[34] Barret E, Sanchez-Salas R, Kasraeian A, Benoist N, Ganatra A, Cathelineau X (2009). A transition to laparoendoscopic single-site surgery (LESS) radical prostatectomy: Human cadaver experimental and initial clinical experience. J Endourol.

[35] Barret E, Sanchez-Salas R, Cathelineau X, Rozet F, Galiano M, Vallancien G (2009). Re: Initial complete laparoendoscopic single-site surgery robotic assisted radical prostatectomy(LESS-RARP). Int Braz J Urol.

[36] Desai MM, Aron M, Canes D, Fareed K, Carmona O, Haber GP (2008). Single-port transvesical simple prostatectomy: Initial clinical report. Urology.

[37] Desai MM, Fareed K, Berger AK, Astigueta JC, Irwin BH, Aron M (2010). Single-port transvesical enucleation of the prostate: A clinical report of 34 cases. BJU Int.

[38] Kaouk JH, Palmer JS (2008). Single-port laparoscopic surgery: Initial experience in children for varicocelectomy. BJU Int.

[39] Rane A, Rao P, Rao P (2008). Single-port-access nephrectomy and other laparoscopic urologic procedures using a novel laparoscopic port (R-port). Urology.

[40] Joseph RA, Goh AC, Cuevas SP, Donovan MA, Kauffman MG, Salas NA (2010). “Chopstick” surgery: A novel technique improves surgeon performance and eliminates arm collision in robotic single-incision laparoscopic surgery. Surg Endosc.

[41] Canes D, Lehman AC, Farritor SM, Oleynikov D, Desai MM (2009). The future of NOTES instrumentation: Flexible robotics and in vivo minirobots. J Endourol.

[42] Shah BC, Buettner SL, Lehman AC, Farritor SM, Oleynikov D (2009). Miniature in vivo robotics and novel robotic surgical platforms. Urol Clin North Am.

